# Progressive tricuspid regurgitation and elevated pressure gradient after transvenous permanent pacemaker implantation

**DOI:** 10.1002/clc.23656

**Published:** 2021-05-26

**Authors:** Wei‐Chieh Lee, Hsiu‐Yu Fang, Huang‐Chung Chen, Yung‐Lung Chen, Tzu‐Hsien Tsai, Kuo‐Li Pan, Yu‐Sheng Lin, Wen‐Hao Liu, Mien‐Cheng Chen

**Affiliations:** ^1^ Institute of Clinical Medicine College of Medicine, National Cheng Kung University Tainan Taiwan; ^2^ Division of Cardiology, Department of Internal Medicine Kaohsiung Chang Gung Memorial Hospital, Chang Gung University College of Medicine Kaohsiung Taiwan; ^3^ Division of Cardiology Chang Gung Memorial Hospital Chiayi Taiwan

**Keywords:** heart failure hospitalization, transvenous permanent pacemaker implantation, tricuspid regurgitation

## Abstract

**Background:**

The association of postimplant tricuspid regurgitation (TR) and heart failure (HF) hospitalization in patients without HF and preexisting abnormal TR and TR pressure gradient (PG) remain unclear.

**Hypothesis:**

This study aimed to explore the clinical outcomes of progressive postimplant TR after permanent pacemaker (PPM) implantation.

**Methods:**

A total of 1670 patients who underwent a single ventricular or dual‐chamber transvenous PPM implantation at our hospital between January 2003 and December 2017 were included in the study. Patients with prior valvular surgery, history of HF, and baseline abnormal TR and TRPG were excluded. Finally, a total of 1075 patients were enrolled in this study. Progressive TR was defined as increased TR grade of ≥2 degrees and TRPG of >30 mmHg after implant.

**Results:**

In 198 (18.4%) patients (group 1) experienced progressive postimplant TR and elevated TRPG, whereas 877 patients (group 2) did not have progressive postimplant TR. Group 1 had larger change in postimplant TRPG (group 1 vs. group 2; 12.8 ± 9.6 mmHg vs. 1.1 ± 7.6 mmHg; p < .001) than group 2. Group 1 had a higher incidence of HF hospitalization compared to group 2 (13.6% vs. 4.7%; p < .001). Preimplant TRPG (HR: 1.075; 95% confidence interval [CI]: 1.032–1.121; p = .001) was an independent predictor of progressive postimplant TR.

**Conclusions:**

After a transvenous ventricular‐based PPM implantation, 18.4% of patients experienced progressive postimplant TR and elevated TRPG. Higher preimplant TRPG was an independent predictor of progressive postimplant TR.

**Abbreviations**
CAD: coronary artery disease
CKD: chronic kidney disease
CRT: cardiac resynchronization therapy
DM: diabetes mellitus
ECG: electrocardiographic
HF: heart failure
ICD: implantable cardioverter defibrillator
LA: left atrial
LVEDV: LV end‐diastolic volume
LVEF: left ventricular ejection fraction
PPM: permanent pacemaker
TR: tricuspid regurgitation
TRPG: tricuspid regurgitation pressure gradient

## 1 INTRODUCTION

In 1959, an endocardial transvenous lead was firstly introduced for permanent cardiac pacing, which has great benefits in reducing cardiac morbidity and mortality related to symptomatic bradycardia.^1^, ^2^ However, the introduction of transvenous right ventricular pacing leads across the tricuspid valve can be associated with the development of tricuspid regurgitation (TR) and elevated tricuspid regurgitation pressure gradient (TRPG). Indeed, the prevalence of TR was increased in patients with transvenous permanent pacemaker (PPM) compared with the general population.^3^ One previous reported that 21.2% of patients developed worsening TR degree after the transvenous lead implantation and a higher rate of worsening TR in patients with implantable cardioverter defibrillator (ICD) lead compared with PPM.^4^ Another study showed that device type and number of leads placed did not affect the worsening degree of postimplant TR.^5^

TR is associated with heart failure (HF) and is related to right ventricular pressure and volume overloading.^6^ In addition, elevated TRPG is associated with poor prognosis in the patients with HF. TR is also an important prognostic factor in patients with moderate or severe mitral regurgitation.^7^ The underlying mechanisms of transvenous cardiac pacing‐related TR is not fully understood. Several mechanisms have been proposed that included a mechanical effect of the lead interfering the motion of the tricuspid leaflets, RV pacing‐induced desynchronization^8^, ^9^ and leads related tricuspid leaflet injury or perforation, entanglement, impingement, or adherence to the tricuspid valve.^8^ One study reported that worsening TR occurred only in the chronic phase over 2 years, whereas another study reported a temporal trend toward increasing TR both acutely and chronically over 4 years after cardiac devices implantation.^5^, ^10^ Therefore, the prevalence of increased degree of postimplant TR remains conflicting. Moreover, the association of postimplant TR and HF hospitalization in patients without HF and preexisting abnormal TR and abnormal TRPG remains unclear. Accordingly, we conducted this study to assess the prevalence of TR after cardiac device implantation and determine its clinical significance on HF hospitalization in a large retrospective cohort after transvenous ventricular‐based PPM implantation.

## 2 MATERIALS AND METHODS

### 2.1 Patient population

A total of 1670 patients who underwent a single ventricular or dual‐chamber transvenous PPM implantation at our hospital between January 2003 and December 2017 were included in this study. Patients with severe valvular heart disease and/or prior valvular surgery, HF and left ventricular ejection fraction (LVEF) <50%, dilated cardiomyopathy, hypertrophic cardiomyopathy, and preexisting abnormal (mild–moderate, moderate or severe) TR and abnormal (>30 mmHg) TRPG, suggestive of possible pulmonary hypertension,^11^ were excluded. Patients without follow‐up records for PPM and without complete follow‐up echocardiography were also excluded (Supplemental Figure S1). Finally, a total of 1075 patients were enrolled in this study and were divided into two groups: group 1 consisted of 198 patients with increased degree of postimplant TR (≥2 degrees) and abnormal TRPG and group 2 consisted of 877 patients without increased degree of postimplant TR and abnormal TRPG.

Patients with dual‐chamber PPM implantation underwent pacing in the dual chamber rate‐adaptive mode, whereas patients with single ventricular PPM implantation underwent pacing in the ventricular‐inhibited rate‐adaptive mode. General demographics, comorbidities, lead positions, pacing QRS durations, pacing percentages, echocardiographic parameters, HF hospitalization, and cardiovascular and all‐cause mortality were compared between the groups.

Baseline electrocardiographic (ECG) and echocardiographic parameters were obtained at nearest to the implant date. After implantation, pacing‐lead locations were determined using anteroposterior, right‐oblique, and left‐oblique views under fluoroscopy. The pacing QRS duration was measured from the surface 12‐lead ECG within 3 days after PPM implantation. Patients visited the outpatient department at regular intervals (3–6 months). PPM records were obtained at regular intervals, and the ventricular pacing percentage was obtained by telemetry.

### 2.2 Ethical statement

This study conformed to the ethical guidelines of the 1975 Declaration of Helsinki and was approved for human research by the institutional review committee of Kaohsiung Chang Gung Memorial Hospital. All patients were informed to be enrolled in our PPM registry when PPM implantation and did not need informed consent due to the retrospective study.

### 2.3 Echocardiography

Echocardiographic parameters, including left atrial (LA) dimension, LVEF, LV end‐diastolic volume (LVEDV), and TR grade/TRPG, were measured using GE Vivid 9 or Philips IE33. LVEF and LVEDV were quantified by the M‐mode and corrected by the two‐dimensional guided biplane Simpson's method of disc measurements. Baseline echocardiography was performed before implantation. Follow‐up echocardiography was performed at 2‐year intervals thereafter in the absence of clinical events or at the onset of HF.

### 2.4 Definition

Progressive TR was defined as increased TR grade of ≥2 degrees and TRPG of >30 mmHg after implant, and prior study showed that pulmonary hypertension was suspected when TRPG at rest >30 mmHg.^11^ Moderate TR (grade III) was defined as a regurgitant jet extending to less than half of the right atrium, whereas severe TR (grade IV) as a jet extending to more than half of the length of the right atrium.^12^ HF hospitalization was defined as the occurrence of HF events according to a New York Heart Association functional class of III or IV in the absence of other alternative diagnoses. HF symptoms were classified as the New York Heart Association functional class II‐IV required medical treatment. Cardiovascular mortality was defined as sudden death related to arrhythmias, HF, and myocardial infarction. All‐cause mortality was defined as death related to any cause, such as sudden death with undefined reasons, natural course, sepsis, malignancy, and cardiovascular disease.

### 2.5 Study end‐points

The primary study endpoint was TR progression (TR grade ≥ 3) and abnormal TRPG levels (PG >30 mmHg). The secondary study end‐points were late‐onset atrial fibrillation, HF hospitalization, sudden death or ventricular tachyarrhythmias, cardiovascular mortality, and all‐cause mortality.

### 2.6 Statistical analysis

Data are presented as mean ± *SD* or numbers (percentages). Clinical characteristics of the study groups were compared using the *t*‐test for continuous variables and Chi‐square test for categorical variables. Kaplan–Meier curve analysis was performed with the log‐rank test for HF hospitalization and progressive TR in both groups during the follow‐up period. Univariable and multivariable Cox regression analyses for HF hospitalization and progressive TR were performed to determine significant determinants. Multivariable Cox regression analysis included a hazard ratio (HR) < 0.100 for HF hospitalization and progressive TR in univariable Cox regression analyses. Statistical analysis was performed using statistical software (SPSS for Windows, Version 22), and a two‐sided p value of <.05 indicated statistical significance.

## 3 RESULTS

### 3.1 Baseline characteristics of the study patients

Baseline characteristics of the study participants are listed in Table 1. During a median 4.9 (interquartile range: 4.7–5.1) years follow‐up, 198 (18.4%) patients (group 1, mean age 72.1 ± 9 years; 59.6% female) experienced progressive postimplant TR, whereas 877 patients (group 2, mean age 71.9 ± 12 years; 49.8% female) did not have progressive postimplant TR. The percentage of female individuals was higher in group 1 than group 2. Additionally, the prevalence of atrial fibrillation (paroxysmal or non‐paroxysmal) was also higher in group 1. A higher percentage of sick sinus syndrome for PPM was noted in group 1 (group 1 vs. group 2; 63.1% vs. 53.9%; p = .022). There was no difference in the distribution of ventricular lead position, pacing QRS duration, ventricular pacing percentage, serum creatinine level and medication used between the two groups.

**TABLE 1:** Baseline characteristics of the study patients

	Group 1 (*N* = 198)	Group 2 (*N* = 877)	p value
General demographics			
Age (years)	72.1 ± 9.4	71.9 ± 11.9	0.830
Female sex (%)	118 (59.6)	437 (49.8)	.015
BMI (kg/m^2^)	24.8 ± 3.9	25.0 ± 3.7	0.606
Risk factors			
Hypertension (%)	150 (75.8)	623 (71.0)	0.190
Diabetes mellitus (%)	68 (34.3)	299 (34.1)	0.934
Hyperlipidemia (%)	41 (20.7)	176 (20.1)	0.845
Prior stroke (%)	27 (13.6)	142 (16.2)	0.449
Atrial fibrillation (%)	74 (37.4)	249 (28.4)	.016
ESRD (%)	15 (7.6)	41 (4.7)	0.110
PAOD (%)	5 (2.5)	18 (2.1)	0.595
CAD (%)	41 (20.7)	154 (17.6)	0.308
CKD stage >3 (%)	45 (22.7)	189 (21.6)	0.704
Indication of PPM			.022
Sick sinus syndrome	125 (63.1)	473 (53.9)	
AV block	73 (36.9)	404 (46.1)	
Lead position			0.474
Lower septum or apex (%)	56 (28.3)	225 (25.7)	
High septum or near RVOT region (%)	142 (71.7)	652 (74.3)	
Pacing QRS duration (msec)	152.7 ± 30.1	150.2 ± 28.1	0.305
>150 mesc (%)	109 (55.1)	451 (51.4)	0.387
Pacing percentage (%)	48.5 ± 43.4	53.9 ± 44.4	0.243
>50%	51 (25.8)	214 (24.4)	0.715
Laboratory examination			
Creatinine (exclude ESRD) (mg/dl)	1.65 ± 1.12	1.50 ± 0.63	0.340
Medication			
ACEI/ARB use (%)	101 (51.3)	439 (50.9)	0.937
β‐blocker use (%)	44 (22.3)	197 (22.8)	0.925
Echocardiographic parameters			
Preimplant			
LA dimension (mm)	37.8 ± 6.6	36.7 ± 6.3	.063
LVEDV (ml)	107.0 ± 31.9	106.9 ± 29.4	0.963
LVEF (%)	69.1 ± 9.0	69.7 ± 8.3	0.437
Average TRPG (mmHg)	23.1 ± 4.9	20.7 ± 6.1	<.001
The duration of follow‐up (years)	4.7 (4.4–5.4)	4.5 (4.2–4.8)	0.610
Postimplant			
LA dimension (mm)	40.0 ± 6.4	37.2 ± 6.0	<.001
LVEDV (ml)	112.3 ± 41.8	111.0 ± 38.3	0.690
LVEF (%)	61.2 ± 13.2	64.2 ± 12.2	.006
<40%	13 (6.6)	26 (3.0)	.020
TR grade			<.001
Severe (%)	21 (10.6)	0 (0)	
Moderate (%)	97 (49.0)	0 (0)	
Mild (%)	80 (40.4)	375 (42.8)	
Trivial or absence (%)	0 (0)	502 (57.2)	
Average TRPG (mmHg)	35.9 ± 9.1	21.8 ± 5.4	<.001
Changes in postimplant TRPG (mmHg)	12.8 ± 9.6	1.1 ± 7.6	<.001

Abbreviations: AV block, atrioventricular block; ACEI, angiotensin‐converting‐enzyme inhibitor; ARB, angiotensin receptor blocker; BMI, body mass index; ESRD, end stage renal disease; CAD, coronary artery disease; PAOD, peripheral arterial occlusive disease; RVOT, right ventricular outflow tract; LA, left atrium; LVEDV, left ventricular end diastolic volume; LVEF, left ventricular ejection fraction; TRPG, tricuspid regurgitation pressure gradient.

*Note*: Data are expressed as mean ± *SD* or as number (percentage).

### 3.2 Preimplant and postimplant echocardiographic parameters of study patients

At preimplant, group 1 had significantly larger LA dimension (group 1 vs. group 2; 37.8 ± 6.6 mm vs. 36.7 ± 6.3 mm; p = .063) and significantly higher average TRPG (group 1 vs. group 2; 23.1 ± 4.9 mmHg vs. 20.7 ± 6.1 mmHg; p < .001) than group 1 (Table 1). The two groups did not differ in LVEDV and LVEF.

**FIGURE 1:**
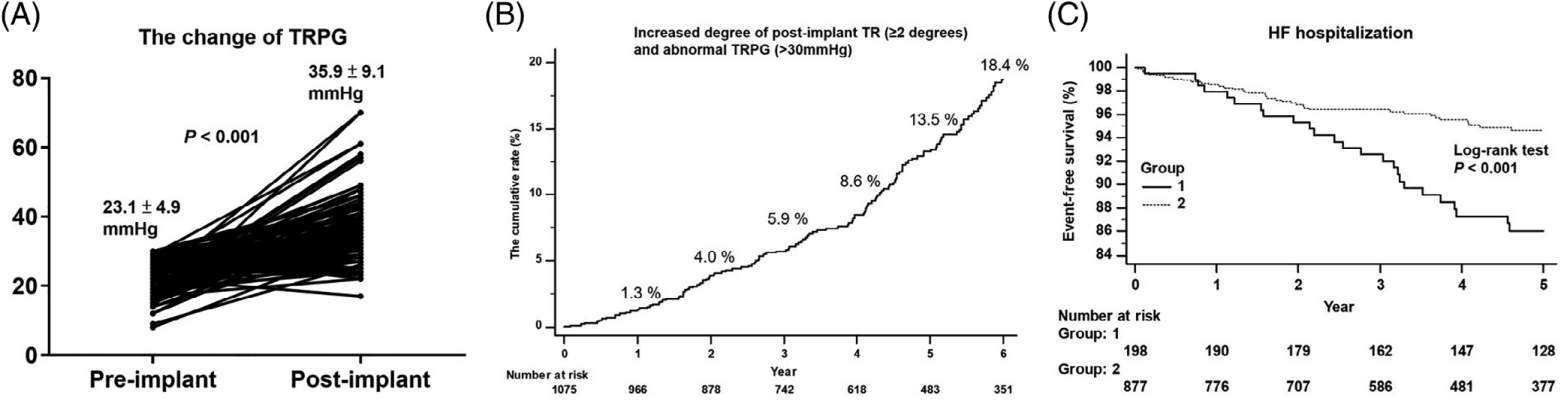
(A) Changes of the tricuspid regurgitation pressure gradient in group 1. In group 1, the postimplant TRPG was significantly higher than preimplant TRPG (p < .001). (B) The cumulative incident rate of progressive postimplant tricuspid regurgitation. The cumulative rate of progressive postimplant TR increased from 1.3% in the first year to 18.4% in the sixth year. (C) A Kaplan–Meier curve analysis for heart failure hospitalization. Group 1 (with progressive postimplant tricuspid regurgitation and elevated tricuspid regurgitation pressure gradient) had a significantly higher incidence of heart failure hospitalization compared to group 2 (without progressive postimplant tricuspid regurgitation and elevated tricuspid regurgitation pressure gradient) (log‐rank p < .001)

The median follow‐up period was similar between the two groups (group 1 vs. group 2; 4.7 (4.4–5.4) years vs. 4.5 (4.2–4.8) years; p = 0.610). At postimplant, group 1 had significantly larger LA dimension, lower LVEF and more severe TR grade than group 2. Additionally, group 1 had significantly higher postimplant TRPG (group 1 vs. group 2; 35.9 ± 9.1 mmHg vs. 21.8 ± 5.4 mmHg; p < .001) and larger changes in postimplant TRPG (group 1 vs. group 2; 12.8 ± 9.6 mmHg vs. 1.1 ± 7.6 mmHg; p < .001) than group 2. Figure 1(A) showed the changes in preimplant and postimplant TRPG in group 1 (p < .001).

Figure 1(B) shows the cumulative incident rate of progressive TR grade and abnormal TRPG from 1.3% in the first year to 18.4% in the sixth year in the study cohort.

### 3.3 Univariable and multivariable Cox regression analyses of predictors of progressive postimplant TR

Female gender, end stage renal disease, atrial fibrillation, larger preimplant LA dimension, and higher preimplant TRPG, were included for multivariable Cox regression analyses of progressive postimplant TR (Table 2). However, only preimplant TRPG (per 1 mmHg increment) (HR: 1.075; 95% confidence interval [CI]: 1.035–1.117; p = .001) was an independent predictor of progressive postimplant TR (Table 2).

**TABLE 2:** Univariable and multivariable Cox regression analyses of predictors of progressive postimplant TR

	Univariable analyses			Multivariable analysis		
Variables	HR	95% CI	p value	HR	95% CI	p value
Female	1.485	1.086–2.031	.013	1.264	0.840–1.904	0.262
Age	1.001	0.988–1.015	0.830			
BMI	0.988	0.946–1.032	0.593			
Diabetes mellitus	1.011	0.731–1.399	0.947			
Hypertension	1.274	0.892–1.819	0.183			
CKD stage of >3	1.071	0.740–1.549	0.717			
ESRD	1.671	0.906–3.084	0.100	1.964	0.893–4.319	.093
Atrial fibrillation	1.505	1.089–2.079	.013	1.280	0.833–1.967	0.260
Late‐onset	1.179	0.533–2.606	0.685			
Coronary artery disease	1.226	0.834–1.802	0.300			
Pacing percentage	0.997	0.992–1.002	0.248			
>50%	0.715	0.477–1.072	0.105			
Pacing QRS length	1.003	0.997–1.009	0.284			
>150 msec	1.172	0.845–1.626	0.342			
V lead position at the lower septum and apex	1.143	0.810–1.613	0.448			
Preimplant LA	1.026	1.000–1.054	.054	1.022	0.990–1.055	0.188
Preimplant LVEDV	1.000	0.995–1.006	0.961			
Preimplant LVEF	0.992	0.972–1.012	0.413			
Preimplant TRPG (per 1 mmHg)	1.078	1.039–1.120	<.001	1.075	1.035–1.117	.001
ACEI/ARB	1.016	0.746–1.385	0.919			
Β‐blocker	0.972	0.671–1.409	0.882			

Abbreviations: ACEI, angiotensin‐converting‐enzyme inhibitor; ARB, angiotensin II receptor *blocker*; BMI, body mass index; CI, confidence interval; CKD, chronic kidney disease; ESRD, end‐stage renal disease; V, ventricular; LA, left atrium; LVEDV, left ventricular end‐diastolic volume; LVEF, left ventricular ejection fraction; TRPG, tricuspid regurgitation pressure gradient; TR, tricuspid regurgitation; OR, odds ratio.

Compared to patients with preimplant TRPG ≤25 mmHg, patients with preimplant TRPG between >25 and ≤ 30 mmHg had 1.825 times (CI: 1.202–2.770; p = .006) of relative risk of developing progressive postimplant TR during follow‐up period.

### 3.4 Clinical outcomes of the study patients

During the follow‐up period, group 1 had a significantly higher incidence of HF hospitalization compared to group 2 (13.6% vs. 4.7%; p < .001) (Table 3 and Figure 1(C). However, the incidence of late‐onset atrial fibrillation, sudden death or ventricular tachyarrhythmias, cardiovascular mortality (group 1 vs. group 2; 5.7% vs. 4.1%; p = .413), and all‐cause mortality (group 1 vs. group 2; 16.2% vs. 14.1%; p = .503) did not differ between the two groups during follow‐up period (Table 3).

**TABLE 3:** Clinical outcomes of the study patients

	Group 1 (*N* = 198)	Group 2 (*N* = 877)	p value
Incidence of late‐onset atrial fibrillation (%)	8 (6.5)	35 (5.6)	0.672
Incidence of HF hospitalization (%)	27 (13.6)	41 (4.7)	<.001
Incidence of sudden death or VTAs (%)	4 (2.1)	23 (2.6)	0.803
Incidence of cardiovascular mortality (%)	10 (5.7)	32 (4.1)	0.413
Incidence of all‐cause mortality (%)	32 (16.2)	124 (14.1)	0.503

Abbreviations: HF, heart failure; VTAs, ventricular tachyarrhythmias.

*Note*: Data are expressed as number (percentage).

### 3.5 Univariable and multivariable Cox regression analyses of predictors of HF hospitalization

By univariable Cox regression analyses, older age, high body mass index, diabetes mellitus (DM), coronary artery disease (CAD), longer pacing QRS length, ventricular lead position at the lower septum and apex, larger preimplant LA dimension, larger preimplant LVEDV, larger postimplant LA dimension, larger postimplant LVEDV, lower postimplant LVEF, postimplant LVEF <40%, and progressive postimplant TR were significant predictors of HF hospitalization (Table 4). However, by multivariable Cox regression analyses, only older age (HR: 1.073; 95% CI: 1.037–1.110; p < .001), chronic kidney disease (CKD) stage of >3 (moderate to severe CKD) (HR: 1.865; 95% CI: 1.008–3.450; p = .047), and larger postimplant LVEDV (HR: 1.010; 95% CI: 1.004–1.017; p = .001) were independent predictors of HF hospitalization. Postimplant LVEF (HR: 0.957; 95% CI: 0.934–0.980; p < .001) was independently inversely associated with HF hospitalization in multivariable analysis. Progressive postimplant TR was significantly associated with HF hospitalization in univariable analysis (HR: 2.459; 95% CT: 1.511–4.000; p < .001) and had a non‐significant trend toward HF hospitalization in multivariable analysis (HR: 1.694; 95% CI: 0.959–2.994; p = .070).

**TABLE 4:** Univariable and multivariable Cox regression analyses of predictors of HF hospitalization

	Univariate analysis			Multivariate analysis		
Variables	HR	95% CI	p value	HR	95% CI	p value
Female	0.951	0.591–1.530	0.836			
Age	1.061	1.032–1.091	<.001	1.073	1.037–1.110	<.001
BMI	1.145	1.082–1.211	<.001			
Diabetes mellitus	2.729	1.692–4.402	<.001			
Hypertension	1.588	0.882–2.859	0.123			
CKD stage of >3	1.634	0.970–2.755	.065	1.865	1.008–3.450	.047
ESRD	1.292	0.405–4.119	0.665			
Atrial fibrillation	0.841	0.490–1.441	0.528			
Late‐onset	0.976	0.303–3.138	0.967			
Coronary artery disease	2.773	1.685–4.562	<.001	1.790	0.986–3.251	.056
Pacing percentage	1.003	0.994–1.012	0.476			
>50%	1.363	0.618–3.002	0.443			
Pacing QRS length	1.013	1.004–1.013	0.008			
>150 msec	1.451	0.858–2.455	0.165			
V lead position at the lower septum and apex	1.812	1.107–2.968	.018	1.713	0.952–3.082	.072
Preimplant LA	1.063	1.024–1.102	.001			
Preimplant LVEDV	1.010	1.002–1.017	.014			
Preimplant LVEF	0.971	0.945–0.999	.042			
Preimplant TRPG	1.020	0.968–1.075	0.448			
Postimplant LA	1.070	1.030–1.111	<.001			
Postimplant LVEDV	1.017	1.013–1.021	<.001	1.010	1.004–1.017	.001
Postimplant LVEF	0.923	0.907–0.940	<.001	0.957	0.934–0.980	<.001
Postimplant LVEF <40%	13.045	7.662–22.209	<.001			
Progressive postimplant TR	2.459	1.511–4.000	<.001	1.694	0.959–2.994	.070
ACEI/ARB	1.600	0.975–2.625	.063			
Β‐blocker	1.329	0.781–2.260	.294			

Abbreviations: ACEI, angiotensin‐converting‐enzyme inhibitor; ARB, angiotensin II receptor *blocker*; BMI, body mass index; CI, confidence interval; CKD, chronic kidney disease; ESRD, end‐stage renal disease; V, ventricular; LA, left atrium; LVEDV, left ventricular end‐diastolic volume; LVEF, left ventricular ejection fraction; TRPG, tricuspid regurgitation pressure gradient; TR, tricuspid regurgitation; OR, odds ratio.

## 4 DISCUSSION

In the present study, the cumulative rate of progressive TR ranged from 1.3% in the first year to 18.4% in the sixth year. Higher preimplant TRPG (per 1 mmHg increment) was positively associated with progressive postimplant TR, which was associated with a trend toward HF hospitalization. Compared to patients with preimplant TRPG ≤25 mmHg, patients with preimplant TRPG between >25 and ≤ 30 mmHg had 1.825 times of relative risk of developing progressive postimplant TR during follow‐up period.

TR occurs mainly due to annular dilation and right ventricular enlargement, often secondary to LV dysfunction from myocardial or valvular causes, right ventricular volume and pressure overload, and cardiac chamber dilations.^13^ Lead‐related TR is an underdetermined problem and may be caused by lead‐related tricuspid leaflet injury or perforation or lead entanglement, impingement, or adherence to the tricuspid valve.^8^ However, lead‐related tricuspid valve injury could not be fully detected and was only observed in 12% of patients with PPM‐related severe TR by transthoracic echocardiography.^8^ Kim et al. reported that abnormal TR developed in 21.2%, worsened TR by ≥1 grade in 24.2%, and progressed to severe TR in 3.9% of patients with initially normal TR.^4^ However, Al‐Bawardy et al. reported a small but significant increase in the prevalence of moderate and severe TR, both acutely and chronically after a cardiac device implantation.^5^ Arabi et al. reported that TR was worsened by 1 grade in 70.8% and 2 grades in 17.1% of patients, and 19.5% of patients without baseline TR developed new‐onset TR after the lead implantation in the follow‐up period.^14^ In this study, the cumulative rate of progressive postimplant TR (increased TR grade of ≥2 degrees and TRPG of >30 mmHg) was from 1.3% in the first year to 18.4% in the sixth year. Moreover, higher pre‐implant TRPG was an independent predictor of progressive postimplant TR. Pacing‐induced electrical and mechanical dyssynchrony of LV can also result in TR and MR.^15^ However, in this study, pacing percentage and pacing QRS length was not associated with the development of progressive postimplant TR. Our previous study showed that right and left atrial sizes were larger in patients with atrioventricular dyssynchrony after pacing.^16^ Atrial enlargement is a well‐known predictor of atrial fibrillation. Utsunomiya et al reported that functional TR with a structurally normal tricuspid valve may occur secondary to chronic atrial fibrillation and is associated with advanced age and right atrial enlargement.^17^ However, atrial fibrillation and LA size were not associated with the development of progressive postimplant TR in our study.

In one retrospective cohort study, significant lead‐induced TR was associated with a significantly increased incidence of all‐cause mortality and HF events in patients after PPM implantation.^18^ Other studies also reported postimplant TR to be an independent risk factor for late death.^5^, ^15^ However, a significant proportion of patients in previous studies included patients with HF and receiving ICD and cardiac resynchronization therapy (CRT). Patients with ICDs and/or CRT devices usually have poor LVEF and advanced HF and consequently, higher incident HF hospitalization and mortality. In our study, we only enrolled patients receiving PPM implantation and excluded patients receiving ICD or CRT and those with prior history of HF, valvular heart disease and preexisting abnormal (mild–moderate, moderate or severe) TR and abnormal (>30 mmHg) TRPG. In this large cohort study, progressive postimplant TR was significantly associated with HF hospitalization in univariable analysis (HR: 2.459; 95% CI: 1.511–4.000; p < .001) and was associated with a non‐significant trend toward HF hospitalization (p = .070) in multivariable analysis, and progressive postimplant TR was not associated with cardiovascular and all‐cause mortality. Therefore, patients with preserved LV function and without valve disease underwent transvenous ventricular‐based pacemaker implantation should have baseline echocardiography evaluation before implant and those with higher preimplant TRPG should have more vigorously echocardiographic follow‐up for the development of progressive postimplant TR.

### 4.1 Study limitations

One limitation of this study is its retrospective nature, including data from only one medical center. Because of older age, the all‐cause mortality rate was relatively high in this study. Another limitation was the absence of baseline and follow‐up right heart size and function by echocardiography. However, we still provided important information about lead‐related postimplant TR progression and its associated outcomes in patients with transvenous ventricular‐based PPM.

## 5 CONCLUSIONS

After a transvenous ventricular‐based PPM implantation, 18.4% of patients experienced progressive postimplant TR and elevated TRPG. Patients with progressive postimplant TR had a higher incidence of HF hospitalization. Higher preimplant TRPG was an independent predictor of progressive postimplant TR.

## CONFLICT OF INTEREST

The author declares there is no potential conflict of interest.

## AUTHORS' CONTRIBUTIONS

Data curation, Huang‐Chung Chen and Hsiu‐Yu Fang; formal analysis, Wei‐Chieh Lee; investigation, Wei‐Chieh Lee; methodology, Wei‐Chieh Lee; project administration, Wei‐Chieh Lee; resources, Yung‐Lung Chen, Tzu‐Hsien Tsai, Kuo‐Li Pan, Yu‐Sheng Lin, Wen‐Hao Liu, Mien‐Cheng Chen; supervision, Mien‐Cheng Chen; validation, Wei‐Chieh Lee; visualization, Wei‐Chieh Lee; writing original draft, Wei‐Chieh Lee; writing, reviewing and editing, Mien‐Cheng Chen. All authors have read and approved the manuscript.

## Supporting information

**Figure S1** Flowchart of the study enrollment. DCM, dilated cardiomyopathy; HF, heart failure; LVEF, left ventricular ejection fraction; TR, tricuspid regurgitation; TRPG, tricuspid regurgitation pressure gradient; TV, tricuspid valve.

## Data Availability

The data that support the findings of this study are available from the corresponding author upon reasonable request.

## References

[1] Furman S, Schwedel J. An intracardiac pacemaker for stokes‐Adams seizures. N Engl J Med. 1959;261:943‐948. 13825713 10.1056/NEJM195911052611904

[2] Akerström F, Pachón M, Puchol A, et al. Chronic right ventricular apical pacing: adverse effects and current therapeutic strategies to minimize them. Int J Cardiol. 2014;173(3):351‐360. 24721486 10.1016/j.ijcard.2014.03.079

[3] Paniagua D, Aldrich HR, Lieberman EH, Lamas GA, Agatston AS. Increased prevalence of significant tricuspid regurgitation in patients with transvenous pacemakers leads. Am J Cardiol. 1998;82(9):1130‐1132. A9. 9817497 10.1016/s0002-9149(98)00567-0

[4] Kim JB, Spevack DM, Tunick PA, et al. The effect of transvenous pacemaker and implantable cardioverter defibrillator lead placement on tricuspid valve function: an observational study. J Am Soc Echocardiogr. 2008;21(3):284‐287. 17604958 10.1016/j.echo.2007.05.022

[5] Al‐Bawardy R, Krishnaswamy A, Rajeswaran J, et al. Tricuspid regurgitation and implantable devices. Pacing Clin Electrophysiol. 2015;38(2):259‐266. 25377489 10.1111/pace.12530

[6] Nath J, Foster E, Heidenreich PA. Impact of tricuspid regurgitation on long‐term survival. J Am Coll Cardiol. 2004;43(3):405‐409. 15013122 10.1016/j.jacc.2003.09.036

[7] Ruel M, Rubens FD, Masters RG, Pipe AL, Bédard P, Mesana TG. Late incidence and predictors of persistent or recurrent heart failure in patients with mitral prosthetic valves. J Thorac Cardiovasc Surg. 2004;128(2):278‐283. 15282466 10.1016/j.jtcvs.2003.11.048

[8] Lin G, Nishimura RA, Connolly HM, Dearani JA, Sundt TM 3rd, Hayes DL. Severe symptomatic tricuspid valve regurgitation due to permanent pacemaker or implantable cardioverter‐defibrillator leads. J Am Coll Cardiol. 2005;45(10):1672‐1675. 15893186 10.1016/j.jacc.2005.02.037

[9] Roeffel S, Bracke F, Meijer A, et al. Transesophageal echocardiographic evaluation of tricuspid valve regurgitation during pacemaker and implantable cardioverter defibrillator lead extraction. Pacing Clin Electrophysiol. 2002;25(11):1583‐1586. 12494615 10.1046/j.1460-9592.2002.01583.x

[10] Webster G, Margossian R, Alexander ME, et al. Impact of transvenous ventricular pacing leads on tricuspid regurgitation in pediatric and congenital heart disease patients. J Interv Card Electrophysiol. 2008;21(1):65‐68. 18040765 10.1007/s10840-007-9183-0 PMC4260457

[11] Ciurzyński M, Bienias P, Irzyk K, et al. Usefulness of echocardiography in the identification of an excessive increase in pulmonary arterial pressure in patients with systemic sclerosis. Kardiol Pol. 2011;69(1):9‐15. 21267956

[12] Polewczyk A, Kutarski A, Tomaszewski A, et al. Lead dependent tricuspid dysfunction: analysis of the mechanism and management in patients referred for transvenous lead extraction. Cardiol J. 2013;20(4):402‐410. 23913459 10.5603/CJ.2013.0099

[13] Rogers JH, Bolling SF. The tricuspid valve: current perspective and evolving management of tricuspid regurgitation. Circulation. 2009;119(20):2718‐2725. 19470900 10.1161/CIRCULATIONAHA.108.842773

[14] Arabi P, Özer N, Ateş AH, Yorgun H, Oto A, Aytemir K. Effects of pacemaker and implantable cardioverter defibrillator electrodes on tricuspid regurgitation and right sided heart functions. Cardiol J. 2015;22(6):637‐644. 26412607 10.5603/CJ.a2015.0060

[15] Höke U, Auger D, Thijssen J, et al. Significant lead‐induced tricuspid regurgitation is associated with poor prognosis at long‐term follow‐up. Heart. 2014;100(12):960‐968. 24449717 10.1136/heartjnl-2013-304673

[16] Lin YS, Guo GB, Chen YL, et al. Atrial enlargement in symptomatic heart block patients with preserved left ventricular function: possibly related to atrioventricular dyssynchrony. Int J Cardiol. 2011;148(3):280‐284. 19942307 10.1016/j.ijcard.2009.11.005

[17] Utsunomiya H, Itabashi Y, Mihara H, et al. Functional tricuspid regurgitation caused by chronic atrial fibrillation: a real‐time 3‐dimensional transesophageal echocardiography study. Circ Cardiovasc Imaging. 2017;10(1):e004897. 28073806 10.1161/CIRCIMAGING.116.004897

[18] Sadeghpour A, Hassanzadeh M, Kyavar M, et al. Impact of severe tricuspid regurgitation on long term survival. Res Cardiovasc Med. 2013;2(3):121‐126. 25478507 10.5812/cardiovascmed.10686 PMC4253772

