# Supplementary figures and images for: Progressive tricuspid regurgitation and elevated pressure gradient after transvenous permanent pacemaker implantation
Source: Clin Cardiol. 2021 May 26;44(8):1098–105. doi: 10.1002/clc.23656 (PMC8364716; doi:10.1002/clc.23656)

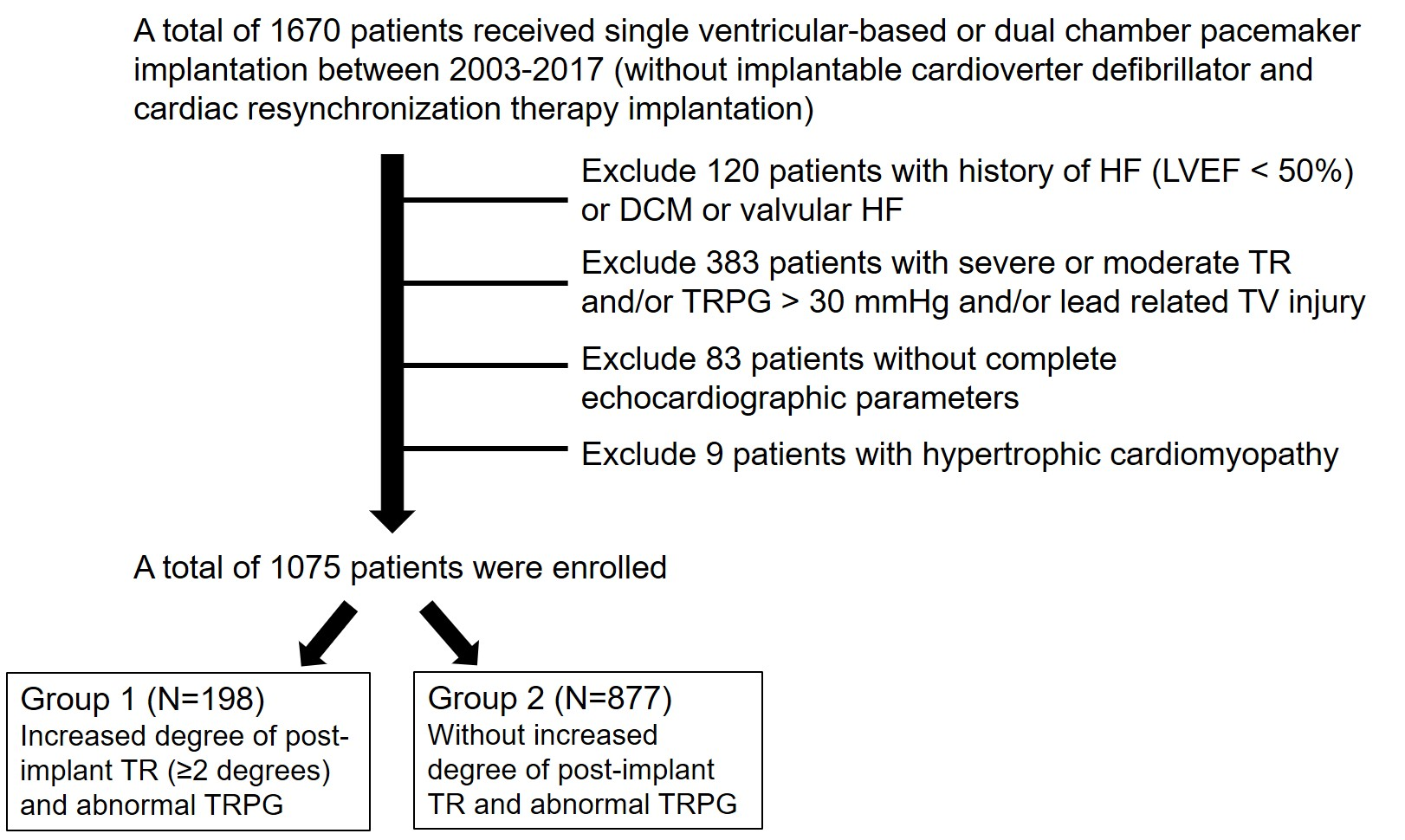

Supplement: Supplementary file 1 — Figure S1 Flowchart of the study enrollment. DCM, dilated cardiomyopathy; HF, heart failure; LVEF, left ventricular ejection fraction; TR, tricuspid regurgitation; TRPG, tricuspid regurgitation pressure gradient; TV, tricuspid valve. [file CLC-44-1098-s001.tif]
